# High-Efficiency and Low-Defect Removal Mechanism of Silicon Carbide Using Center-Inlet Computer-Controlled Polishing

**DOI:** 10.3390/mi17030298

**Published:** 2026-02-27

**Authors:** Pengli Lei, Baojian Ji, Jing Hou, Mincai Liu, Wenhui Deng, Fei Fan, Jian Wang, Bo Zhong

**Affiliations:** Laser Fusion Research Center, China Academy of Engineering Physics, Mianyang 621900, China

**Keywords:** reaction bonded silicon carbide, center-inlet polishing, high efficiency, low defects

## Abstract

Reaction-bonded silicon carbide (RB-SiC) is the preferred material for space optical systems because of its low density and high specific stiffness. However, its hardness and multi-component properties lead to low efficiency and pit defects during the polishing process, making the fabrication of RB-SiC a significant challenge. This study proposes a high-efficiency and low-defect fabrication method for RB-SiC using center-inlet computer-controlled polishing (CCP). We first investigated the polishing efficiency and surface quality achieved with center-inlet and non-center-inlet liquids. The results show that the defect density under non-center-inlet conditions was positively correlated with process parameters, while fewer defects and higher efficiency could be achieved under center-inlet conditions. Additionally, the efficient removal and defect suppression mechanisms under the center-inlet condition were revealed based on machining force, heat, and defect characterization. Under center-inlet conditions, the friction coefficient is larger and stable, resulting in high removal efficiency. The macro–micro coupled analysis results show that pit defects are generated through the combined action of force and heat, which leads to the thermo-mechanical degradation and shedding of SiC particles due to the temperature increase in the machining zone. The results demonstrate that center-inlet CCP not only ensures sufficient abrasion at the polishing interface to achieve high removal efficiency but also significantly suppresses the processing heat, thereby resulting in a low-defect surface.

## 1. Introduction

Space science and technology advancements have driven substantial improvements in the performance of optical systems in space applications. Accordingly, requirements for the operational wavelength range, imaging resolution, thermal stability, and overall system mass have become more rigorous, and the development of large-aperture, lightweight, and reflective optical system architectures has accelerated [1,2,3]. Due to their advantageous properties—including their low density, high modulus, high specific stiffness, superior dimensional stability, and favorable thermal characteristics—silicon carbide (SiC) materials have become the preferred choice for next-generation space-based mirrors [4]. Large-aperture SiC aspheric mirrors are currently employed in a variety of advanced systems, such as spaceborne optical remote sensing detectors, high-resolution Earth observation platforms, and space telescopes. Notable examples include the SPICA infrared astronomical telescope [5], the ASTRO-F astronomical telescope [6], and the James Webb Space Telescope (JWST) [7].

Compared to traditional optical glass, SiC material presents challenges such as high hardness and multi-component characteristics, making it difficult to achieve efficient, high-precision, and ultra-smooth processing of SiC mirrors [8]. In particular, processing large, lightweight, and shaped RB-SiC silicon carbide aspheric mirrors is challenging and time-consuming. The main difficulties lie in defect suppression and low removal efficiency. Numerous scholars have explored the optical processing of RB-SiC mirrors in recent years, gaining significant experience in enhancing efficiency and suppressing defects. Regarding defect suppression, T. Nguyen et al. [9] studied the wear mechanism of reactively bonded silicon carbide under mixed abrasive polishing and loose abrasive water jet (AWJ) impact conditions. The material’s wear characteristics are distinguished by different mechanisms involving its silicon and silicon carbide components. Wear primarily occurs through erosion and wedging, which weaken the Si bonds and ultimately lead to the release of SiC particles from the material structure. It was found that RB-SiC can be processed with a relatively low-pressure alumina slurry jet without inflicting any surface damage. Deng et al. [10] reported a hybrid polishing process combining thermal oxidation pretreatment and soft abrasive polishing to achieve damage-free and atomically smooth polishing of 4H-SiC carbon surfaces. Chen et al. [11] proposed a novel non-resonant vibration-assisted roll polishing (NVRP) technique to process silicon carbide ceramic workpieces. Tama et al. [12] presented a study that focused on the efficient fabrication of RB-SiC optical components using a plate fixed with solid pellet-shaped abrasives for rapid removal of surface errors and initial smoothing.

Wang et al. [13] reported a micro-interaction involving fabricating RB-SiC material with a fixed abrasive. They quantitatively discussed the influence of the depth formed on the RB-SiC workpiece by the diamond abrasive on the material removal rate and the surface roughness of an optical component. Considering the low polishing efficiency and quality due to its extreme hardness, Chen et al. [14] developed an ultrasonic chemical-assisted polishing (UCAP) approach for SiC using hybrid hydroxyl/diamond slurries and investigated its polishing characteristics and mechanisms. Agarwal et al. [15] investigated a high-removal-rate grinding method for silicon carbide with respect to material removal and basic grinding parameters. Furthermore, Gu et al. [16] established a finite element analysis model of the polishing process of silicon carbide ceramics and studied the change processes of the polishing force, which they suggested can lead to the selection of reasonable polishing parameters to obtain good surface quality.

In summary, it is evident that advanced manufacturing techniques such as loose abrasive water jet polishing (AWJ), non-resonant vibration-assisted roll-type polishing, and ultrasonic chemical-assisted polishing have significantly enhanced the production efficiency and surface quality of silicon carbide materials. However, there is still little research on the application of these methods to meter-scale or larger silicon carbide optical components. The fabrication processes for large-aperture elements are still constrained due to challenges in developing damage-free and cost-effective machining techniques, which are yet to be perfected. These limitations prevent them from meeting the rapid deployment needs of space optical systems, where there is often a desire to escalate the machining rate to boost productivity while preserving the required surface integrity. This study introduces an innovative high-efficiency, low-defect manufacturing method for silicon carbide based on center-inlet fluid and systematically explores the interconnected relationships between “process parameters, processing force/heat, efficiency, and defects,” providing a fundamental understanding of the material removal and damage formation mechanisms under center-inlet CCP conditions. Crucial process factors for superior silicon carbide processing are defined, offering vital technical support for the ultra-precision manufacturing of large-caliber RB-SiC mirrors.

## 2. Method

### 2.1. Experimental Setup

**Figure 1:**
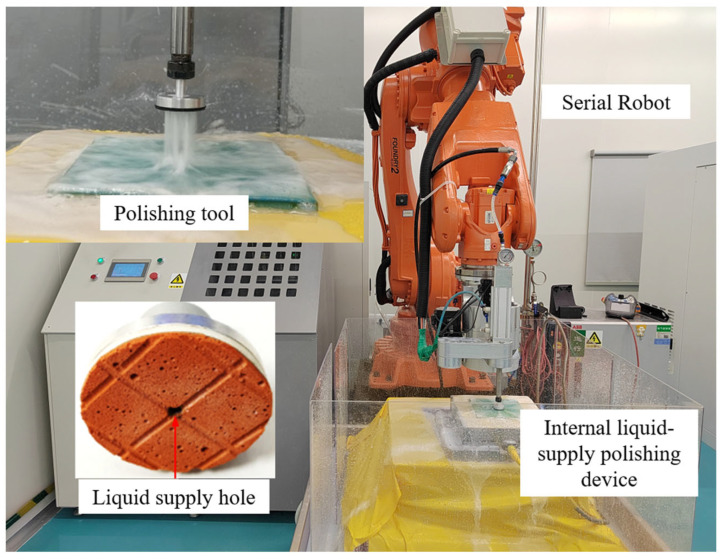
Center-inlet polishing unit with hollow tool.

A novel center-inlet CCP process and tool has previously been presented for the high-efficiency polishing of large-diameter aspheric optics [17]. The most distinctive feature of center-inlet CCP is that the polishing slurry is supplied by the center-inlet polishing tool, which constitutes an internal supply method, as illustrated in Figure 1. In contrast, the polishing slurry in traditional CCP is delivered externally. This study introduces an improved center-inlet CCP technique that integrates the capabilities of high-pressure polishing fluid supply and in situ force measurement. We constructed a center-inlet polishing system built around a high-pressure fluid delivery apparatus, a hollow tool, a force sensor, and an industrial robot.

### 2.2. Removal Function and Polishing Force Acquisition Experiment

To analyze the removal characteristics of different liquid supply methods, spot experiments were conducted using both internal and external fluid supplies at various rotational speeds: 200, 500, 1000, and 1500 rpm. Other experimental conditions included a polishing fluid particle size of 3 μm, an air pressure of 0.15 MPa, an orbital speed of 200 rpm, and a polishing time of 300 s. During the experiments, a force sensor was positioned beneath the component to measure the polishing forces under different process conditions. The specific experimental conditions are outlined in Table 1.

### 2.3. Processing Defects and Thermal Inspection

#### Comparison of Material Removal Characteristics

**Figure 2:**
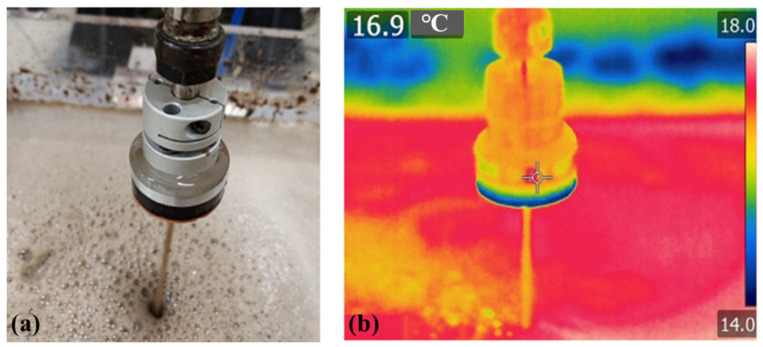
Physical photographs and thermograms of the center-inlet tool in the unmachined state. (**a**) Physical drawing of the center-inlet fluid; (**b**) temperature measurement of the center-inlet fluid.

To address the issue of high defect density in silicon carbide components processed via traditional chemical mechanical polishing, a systematic rapid polishing experiment was conducted using dual liquid supply pathways (internal and external). The experimental parameters included air pressures of 0.10, 0.15, and 0.20 MPa and rotational speeds of 500, 1000, and 1500 rpm, as detailed in Table 2. During the experiment, the temperature of the polishing area was monitored in real time using an infrared thermal imager, as shown in Figure 2. After polishing, the defect density on the component surfaces was statistically analyzed, the defect morphology was characterized microscopically, and the surface roughness was measured with a white light interferometer.

## 3. Results

### 3.1. Morphology and Efficiency of Removal Function

**Figure 3:**
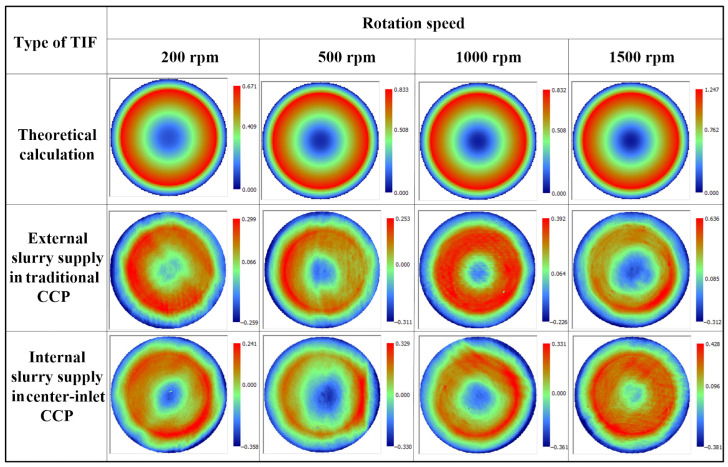
TIF diagrams of the theoretical calculations and the internal and external slurry supply experiments.

To solve the problem of low efficiency in the traditional CCP of RB-SiC, center-inlet CCP with internal slurry supply was applied to quickly polish RB-SiC components. TIF experiments were carried out based on center-inlet and traditional CCP. Figure 3 shows a comparison of the theoretical TIF calculations with internal and external slurry supply.

**Figure 4:**
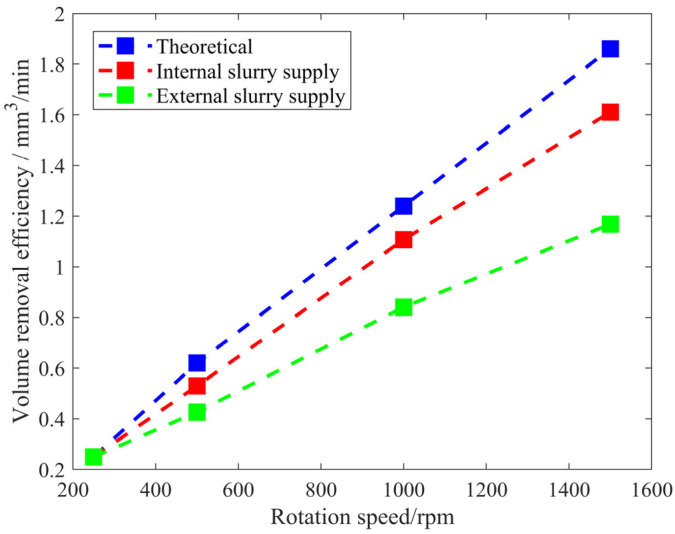
Removal efficiency curves from the theoretical calculations and the internal and external slurry supply experiments.

Figure 4 shows the removal efficiency curves obtained in the experiments conducted with internal and external slurry supply. The theoretical and experimental efficiency are the same at low speed, but the experimental efficiency is lower than theoretical efficiency at high speed, especially with an external slurry supply. With an increase in the rotation speed, the removal efficiency under the internal and external slurry supply methods increases approximately linearly, but the increase rate with the internal slurry supply is greater. At high speeds, the efficiency of the internal slurry supply is significantly higher than that of the external slurry supply; for example, at 1500 rpm, the processing efficiency of the internal slurry supply increased by 38% compared with that of the external slurry supply. In this study, the material removal through full-surface polishing is essentially the convolution of the removal function (sometimes referred to as the polishing spot) with the dwell time, which is why researchers have paid considerable attention to the removal function. We observed that the polishing spot obtained using the center-inlet CCP method achieves 38% higher material removal than that obtained with the traditional internal fluid supply method. Based on the linear convolution property of the removal model, it can be theoretically inferred that if the same dwell time distribution is applied in full-surface polishing, the total amount of material removed from the entire meter-scale workpiece would also be 38% higher when using the center-inlet CCP method.

### 3.2. Polishing Force

**Figure 5:**
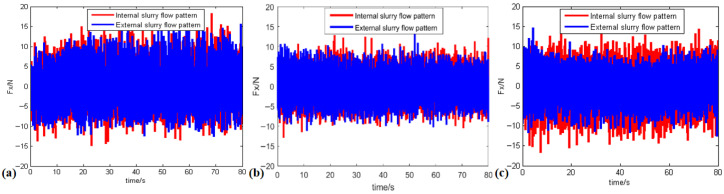
Inner and outer fluid supply friction at different speeds: (**a**) 500 rpm; (**b**) 1000 rpm; (**c**) 1500 rpm.

By measuring normal and tangential forces during single-spot polishing, we analyzed the impacts of various rotational speeds and liquid supply methods on the friction coefficient, thereby elucidating the causes of removal rate discrepancies under different liquid supply conditions. The experimental parameters include rotational speeds of 500, 1000, and 1500 rpm, with additional parameters being a pressure of 0.15 MPa, a detection time of 80 s, and an abrasive particle size of 3 μm. The results of the polishing force acquisition experiments are presented in Table 3 and Figure 5.

### 3.3. Density of Defects

**Figure 6:**
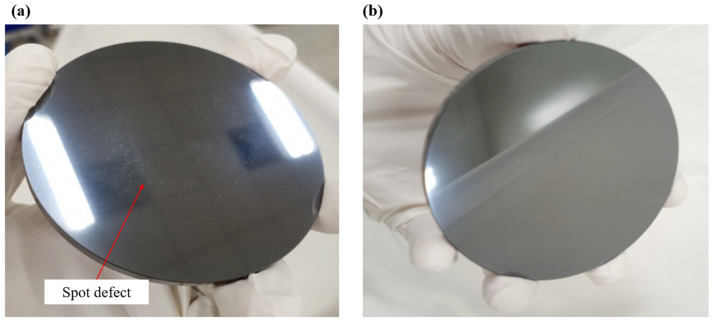
Surface defect diagrams under different liquid supply methods: (**a**) external liquid supply; (**b**) internal liquid supply. Reproduced with permission from Zhong et al., 2023 [18].

**Figure 7:**
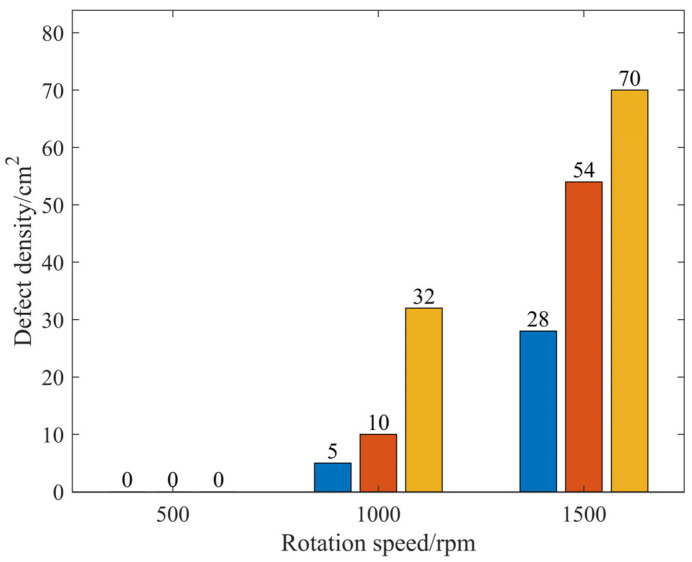
Defect density under different parameters of external liquid supply.

**Figure 8:**
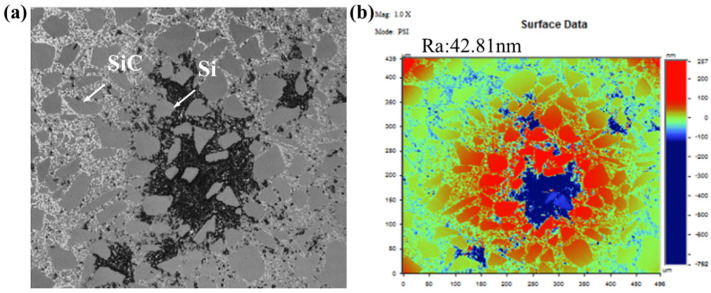
Micro-imaging of SiC surface with external liquid supply. (**a**) Micrograph; (**b**) roughness. Reproduced with permission from Zhong et al., 2023 [18].

**Figure 9:**
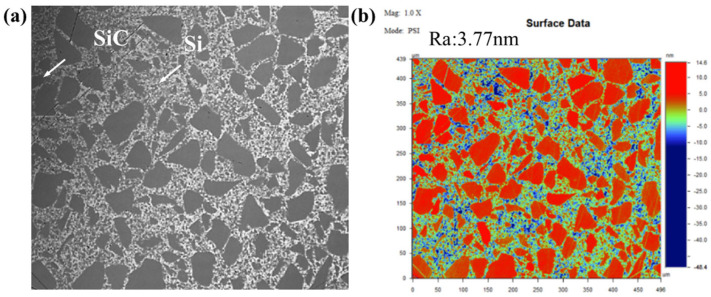
Micro-imaging of SiC surface with internal liquid supply. (**a**) Micrograph; (**b**) roughness. Reproduced with permission from Zhong et al., 2023 [18].

Following the experiment, statistical analysis was performed to determine the defect density across components processed under varying parameters. The morphological features of surface defects were examined using microscopy, while component surface roughness was assessed with a white light interferometer. The corresponding results are presented in Figure 6, Figure 7, Figure 8 and Figure 9. Figure 6 shows a surface quality comparison under different liquid supply modes. Under external liquid supply, a “white spot” defect is observed near the center of the component. In contrast, the internal liquid supply method produces a surface free of such defects.

Figure 7 illustrates the defect density under various parameters with external liquid supply. The results indicate that the defects on the silicon carbide surface correlate with the process parameters when using the external liquid supply, with higher rotational speeds and increased air pressure resulting in a greater defect density on the silicon carbide surface. In contrast, under internal liquid supply conditions, no surface defects on the silicon carbide were observed regardless of the process parameters employed. This highlights the significant advantage of rapid internal liquid supply in substantially suppressing defects.

Figure 8 and Figure 9 display microscopic images of SiC component surfaces processed with internal and external liquid supply methods, respectively. RB-SiC, a composite material consisting of silicon carbide particles within a silicon binder matrix, exhibits a random distribution of both phases in low-defect regions, as shown in Figure 9a. In contrast, surfaces containing defects present signs of thermo-mechanical degradation, as illustrated in Figure 8a. Further evaluation of surface roughness indicates that the observed “white spot” defects correspond to surface pits, with the affected area showing an Rq value of 70.6 nm (Figure 8b). Conversely, low-defect regions demonstrate significantly smoother surfaces, achieving an Rq value of 4.45 nm, as presented in Figure 9b.

### 3.4. Process Heat

**Figure 10:**
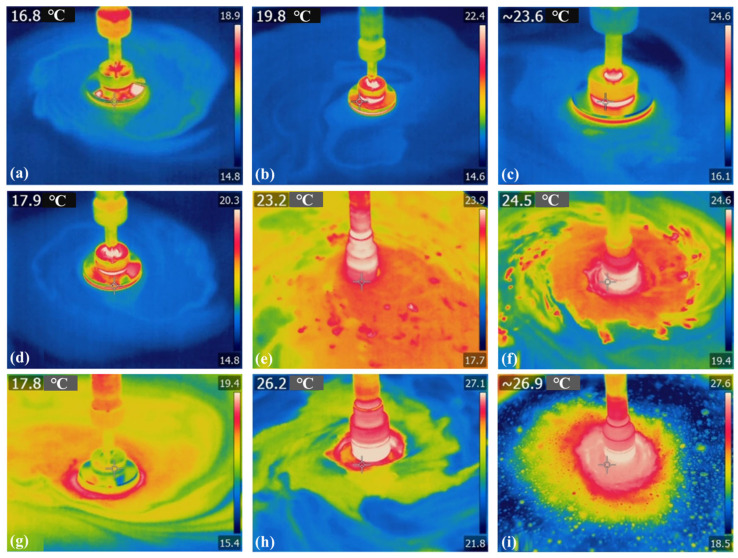
Temperature of processing area under external liquid supply conditions: (**a**) 0.1 MPa, 500 RPM; (**b**) 0.1 MPa, 1000 RPM; (**c**) 0.1 MPa, 1500 RPM; (**d**) 0.15 MPa, 500 RPM; (**e**) 0.15 MPa, 1000 RPM; (**f**) 0.15 MPa, 1500 RPM; (**g**) 0.2 MPa, 500 RPM; (**h**) 0.2 MPa, 1000 RPM; (**i**) 0.2 MPa, 1500 RPM. (**c**) was reproduced with permission from Zhong et al., 2023 [18].

**Figure 11:**
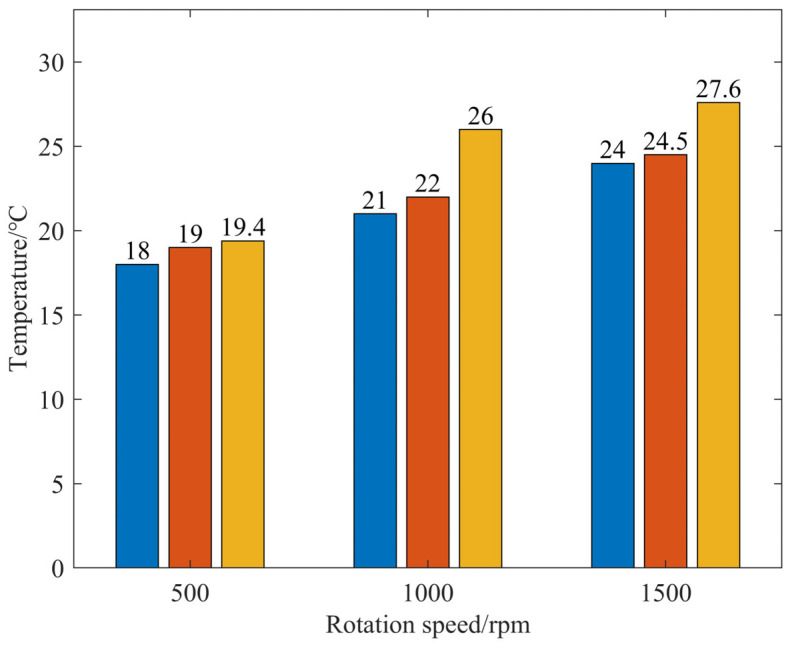
Temperature of the processing zone under external liquid supply conditions.

**Figure 12:**
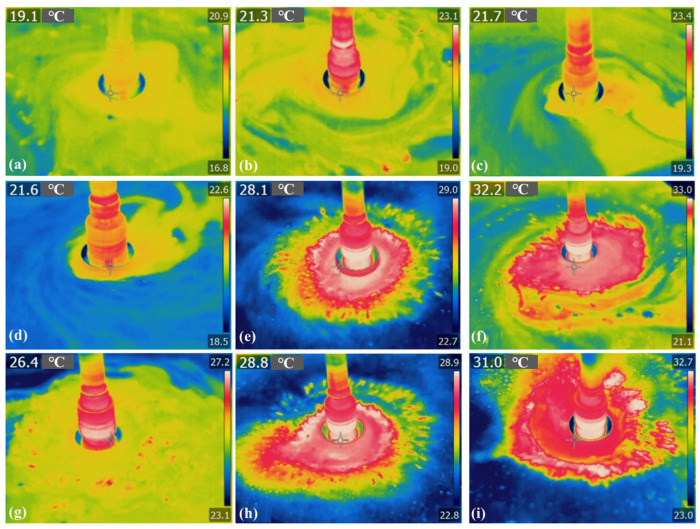
Temperature of processing area under internal fluid supply conditions: (**a**) 0.1 MPa, 500 RPM; (**b**) 0.1 MPa, 1000 RPM; (**c**) 0.1 MPa, 1500 RPM; (**d**) 0.15 MPa, 500 RPM; (**e**) 0.15 MPa, 1000 RPM; (**f**) 0.15 MPa, 1500 RPM; (**g**) 0.2 MPa, 500 RPM; (**h**) 0.2 MPa, 1000 RPM; (**i**) 0.2 MPa, 1500 RPM. (**a**) was reproduced with permission from Zhong et al., 2023 [18].

**Figure 13:**
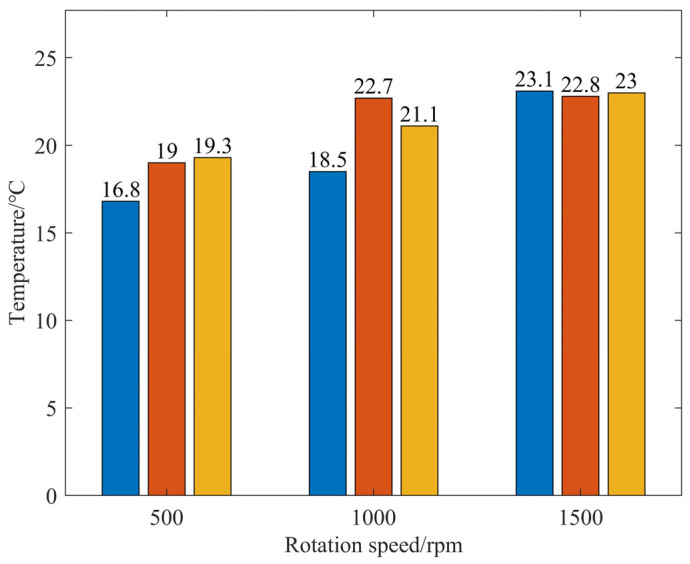
Temperature of the processing zone under internal fluid supply conditions.

We analyzed the processing heat during the single-spot polishing process and evaluated the impacts of different rotational speeds and pressure parameters on the processing heat under both internal and external liquid supply conditions. The experimental parameters included polishing air pressures of 0.1, 0.15, and 0.2 MPa and polishing speeds of 500, 1000, and 1500 rpm. The results of the polishing heat acquisition experiments are presented in Figure 10, Figure 11, Figure 12 and Figure 13.

Figure 10 displays the temperature measurement diagram of the processing area with external fluid supply under various parameters, while Figure 11 shows the corresponding temperature statistics for the processing area. The processing temperature is 18 °C under the conditions of 500 rpm and 0.1 MPa air pressure. In contrast, at 1500 rpm and 0.2 MPa air pressure, the processing temperature reaches 27.6 °C, marking an increase of 53.3%. With external fluid supply, there is notable processing heat at the tool–workpiece contact interface, and both rotational speed and air pressure significantly elevate the temperature in the processing area.

Figure 12 presents a temperature measurement graph of the processing area with internal fluid supply under various parameters, while Figure 13 displays the corresponding temperature statistics for the processing area. The processing temperature is 16.8 °C under the conditions of 500 rpm and 0.1 MPa air pressure; in contrast, at 1500 rpm and 0.2 MPa air pressure, the processing temperature is 23 °C, marking an increase of 36.9%. Under internal fluid supply conditions, there is no significant temperature rise at the tool–workpiece contact interface, and it can be noted that the processing area is actively cooled by the low-temperature polishing fluid. Additionally, an increase in rotational speed does not significantly change the temperature in the center zone of the polishing tool.

The temperature measurements reveal the flow dynamics and replenishment efficiency of the polishing fluid within the contact zone. In the case of external liquid supply, delayed fluid renewal impedes the dissipation of frictional heat generated between the tool and the workpiece at the center of the contact area. This suggests that, under high-speed and high-pressure processing conditions, the external supply method is inadequate for effectively delivering polishing fluid to the central region. Conversely, internal liquid supply ensures timely fluid refreshment, thereby facilitating efficient removal of polishing-induced heat.

Furthermore, the temperature of the polishing fluid was controlled as a constant at 20 °C, with a precision of ±1 °C. The fluid flow rate was maintained at ≥10 L/min, monitored using a digital flow meter. The polishing fluid is typically kept at room temperature to avoid introducing additional thermal disturbances, maintain the stability of the removal function, and effectively suppress polishing quality degradation caused by thermal stress or changes in the physicochemical properties of the fluid. Room temperature conditions not only ensure the controllability of processing heat but also simplify the process system, meeting high surface quality requirements in most precision polishing scenarios. Active control of polishing fluid temperature is only implemented in cases with specific demands for ultra-high precision or efficiency. This study focuses on the comparative analysis between external fluid supply CCP and center-inlet CCP. As one of the controlled experimental conditions, the polishing fluid temperature was held constant. Future work should thoroughly investigate how fluid temperature itself affects the processing heat and defect suppression.

## 4. Discussion

### 4.1. Efficient Removal Mechanism for Rapid Polishing of SiC Materials with Center-Inlet Solution

**Figure 14:**
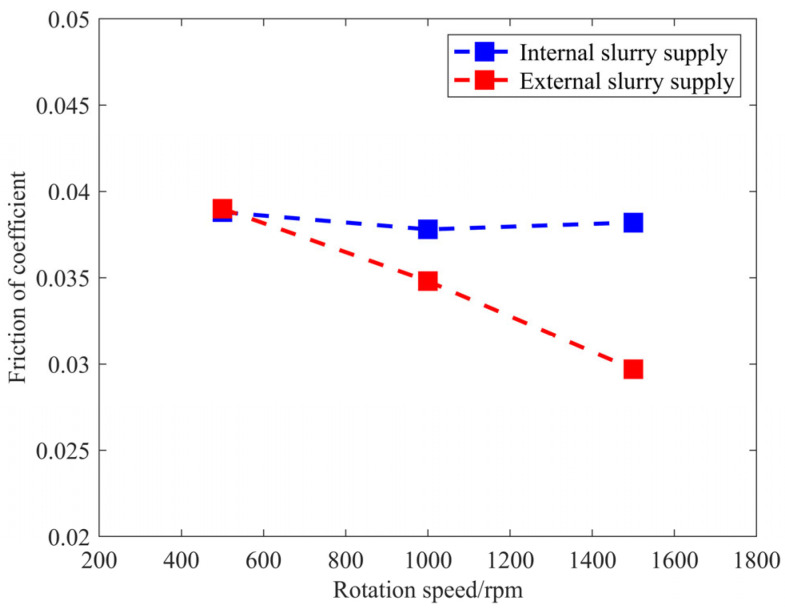
Friction coefficient versus rotational speed for internal and external fluid supply conditions.

According to the experimental results presented in Table 3, we established the relationship between the friction coefficient and rotational speed under both internal and external fluid supply conditions, as depicted in Figure 14. Under external fluid supply conditions, there is a significant reduction in the friction coefficient with an increase in the rotational speed. Specifically, when the rotational speed increases from 500 rpm to 1500 rpm, the friction coefficient decreases from 0.0037 to 0.00242, representing a reduction of approximately 34.6%. In contrast, under internal fluid supply conditions, the friction coefficient remains essentially unchanged with increasing rotational speed.

According to the polishing efficiency results shown in Figure 4, both internal and external fluid supply exhibit an approximately linear increase in efficiency with rotational speed. However, the growth coefficient for internal fluid supply (1  × 10^−4^) is larger than that under an external fluid supply (7 × 10^−5^). In the well-known Preston equation (*R* = *KPV*), the coefficient *K* is related to the state of the polishing contact zone (e.g., the amount of polishing particles, performance of the polishing pad), *P* represents the normal pressure exerted by the tool on the workpiece, and *V* is the relative velocity. In this study, the unit of *R* is expressed as mm^3^/min. The calculated value of *K* is usually the ratio of the experimentally measured removal efficiency *R* to the theoretical prediction *P*·*V*. Strictly speaking, *K* may be a variable with slight fluctuations, but for the sake of computational simplicity, it is widely accepted as a constant [19]. If *V* increases approximately linearly, then *R* also increases approximately linearly. Under external fluid supply conditions, as the friction coefficient decreases with rotational speed, the growth rate of *R* is reduced. Conversely, under internal fluid supply conditions, since the friction coefficient remains essentially unchanged, *R* increases approximately linearly with *V*, and the growth rate is close to theoretical predictions.

### 4.2. Mechanism of Defect Generation in Center-Inlet Rapid Polishing of SiC Materials

**Figure 15:**
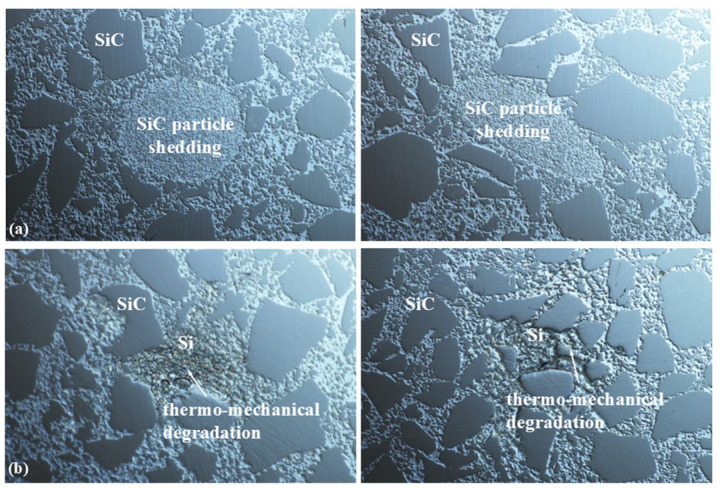
Detection results of pit defects on the surface of silicon carbide. (**a**) The phenomenon of silicon carbide particle shedding; (**b**) surface thermo-mechanical degradation.

According to the experimental results, the removal characteristics of RB-SiC material are influenced by the different mechanisms in its silicon and silicon carbide constituents, primarily manifested in two ways: abrasive particle wear and SiC particle shedding. Abrasive particle wear is caused by the cutting action of abrasive particles on the material surface. Particle shedding occurs because the Si phase in RB-SiC is dispersed among the SiC phases. Thus, abrasive particle erosion or wedging during processing weakens Si bonds, thereby releasing SiC particles from the material structure. The abrasive particle wear removal mechanism is demonstrated by the appearance of plastic cutting textures on the SiC phase on surfaces polished with diamond abrasives. The particle shedding mechanism is demonstrated by a decrease in the bonding strength between the silicon carbide and the matrix under the combined action of thermal and mechanical stresses, which leads to the detachment of silicon carbide particles, as shown in Figure 15a. Besides these two phenomena, thermo-mechanical degradation of the localized Si phase was observed on the surface of the component due to processing heat, as shown in Figure 15b. Through roughness detection, both SiC particle shedding and thermo-mechanical degradation of the Si phase led to pit defects on the surface of the component, appearing as “white spots” on the surface, as shown in Figure 6.

Based on the comprehensive analysis of the interrelationships between force, heat, and defects under the two liquid supply methods discussed in this paper, it is evident that under external liquid supply conditions, the processing heat significantly increases as the force and rotational speed increase, leading to a higher likelihood of surface thermo-mechanical degradation. Simultaneously, the interfacial bonding strength decreases, making the particles susceptible to detachment when subjected to external forces. Compared to external liquid supply processing, internal liquid supply processing exhibits slightly higher friction under the same parameters, with the most significant difference being the processing heat. Under high-speed and high-pressure machining parameters, no defects were observed in the internal liquid supply method, indicating that machining heat is an important factor affecting the interfacial bond strength and the generation of pit defects. This suggests that the center-inlet processing method is an effective means of suppressing the low-defect processing of RB-SiC. Additionally, the size of abrasive particles in the polishing slurry influences the polishing performance, primarily affecting surface roughness and the material removal rate. However, this study specifically compares the polishing performance (e.g., polishing efficiency and surface damage) of center-inlet computer-controlled polishing (CCP) against that under the non-center-inlet configuration. Therefore, it is essential to maintain identical experimental conditions across all tests. The relevant literature has demonstrated that abrasive particle sizes in the range of 1~5 μm are commonly preferred [20]. Furthermore, based on established practices in the field of ultra-precision polishing [19,21], we chose a particle size of 3 μm for our experiments (see Section 2.2 and Section 2.3).

## 5. Conclusions

To improve the processing efficiency and defects of RB-SiC, a high-speed and high-pressure center-inlet CCP approach was proposed, and its high-efficiency and low-defect removal mechanisms for RB-SiC were revealed. The conclusions are as follows.

(1) The removal efficiency of center-inlet CCP is higher than that of traditional CCP under high rotation speeds, as the friction coefficient of center-inlet CCP is larger. The removal efficiency of center-inlet CCP is increased by 38% compared with traditional CCP at a rotation speed of 1500 rpm. In traditional CCP, the friction coefficient is significantly reduced with increased speed while, in center-inlet CCP, the friction coefficient is basically invariant with speed variations.
(2) Higher speeds and pressures lead to a greater density of defects under traditional CCP conditions, while center-inlet CCP produces no defects under any condition. The generation mechanism of pit defects is the thermo-mechanical degradation and shedding of SiC particles caused by the combined action of heat and force.
(3) The temperature rise in the processing zone is the main cause of defects. Center-inlet CCP can significantly suppress the processing heat, thereby obtaining a low-defect surface with excellent surface roughness.
(4) The high-efficiency and low-defect manufacturing of RB-SiC using high-speed and high-pressure center-inlet CCP was realized, providing another potential technical option for the production of RB-SiC mirrors.

## Figures and Tables

**Table 1:** Experimental conditions.

Test Number	Liquid Supply Method	Granularity μm	Pneumatic Mpa	Rotation Speed RPM	Orbital Speed RPM	Time s	Component Number
1	Internal fluid supply	3	0.15	200	200	300	JC1811-41-#9-B
2		3	0.15	500	200	300	
3		3	0.15	1000	200	300	
4		3	0.15	1500	200	300	
5	External fluid supply	3	0.15	200	200	300	JC1811-41-#10-B
6		3	0.15	500	200	300	
7		3	0.15	1000	200	300	
8		3	0.15	1500	200	300	

**Table 2:** Experimental conditions for defect and heat acquisition.

Test Number	Component Number	Granularity μm	Pitch of Spiral mm	Feed Speed mm/min	Pneumatic Mpa	Rotation Speed RPM
1	JC1811-40-#1	3	5	200	0.1	500
2	JC1811-40-#2	3	5	200	0.1	1000
3	JC1811-40-#3	3	5	200	0.1	1500
4	JC1811-40-#4	3	5	200	0.15	500
5	JC1811-40-#5	3	5	200	0.15	1000
6	JC1811-40-#6	3	5	200	0.15	1500
7	JC1811-40-#7	3	5	200	0.2	500
8	JC1811-40-#8	3	5	200	0.2	1000
9	JC1811-40-#9	3	5	200	0.2	1500

**Table 3:** Results of polishing force acquisition experiments.

Flow Pattern	Rotation Speed/rpm								
	500			1000			1500		
	Frictional Force/N	Normal Force/N	Frictional Coefficient	Frictional Force/N	Normal Force/N	Frictional Coefficient	Frictional Force/N	Normal Force/N	Frictional Coefficient
Internal	3.52	90.67	0.0388	3.4	90	0.00378	3.425	89.77	0.00382
External	3.62	92.82	0.0390	3.1	89	0.00348	2.657	89.55	0.00297

## Data Availability

The original contributions presented in this study are included in the article. Further inquiries can be directed to the corresponding author.
